# Inpainting the Neural Picture: Inferring Unrecorded Brain Area Dynamics from Multi-Animal Datasets

**Published:** 2025-10-13

**Authors:** Ji Xia, Yizi Zhang, Shuqi Wang, Genevera I. Allen, Liam Paninski, Cole Lincoln Hurwitz, Kenneth D. Miller

**Affiliations:** Center for Theoretical Neuroscience, Zuckerman Mind Brain Behavior Institute, Kavli Institute for Brain Science, Columbia University; Department of Statistics, Center for Theoretical Neuroscience, Zuckerman Mind Brain Behavior Institute, Kavli Institute for Brain Science, Grossman Center for the Statistics of Mind, Columbia University; Laboratory of Computational Neuroscience, École polytechnique fédérale de Lausanne (EPFL); Department of Statistics, Irving Center for Cancer Dynamics, Center for Theoretical Neuroscience, Zuckerman Mind Brain Behavior Institute, Kavli Institute for Brain Science, Columbia University; Department of Statistics, Center for Theoretical Neuroscience, Zuckerman Mind Brain Behavior Institute, Kavli Institute for Brain Science, Grossman Center for the Statistics of Mind, Columbia University; Center for Theoretical Neuroscience, Zuckerman Mind Brain Behavior Institute, Kavli Institute for Brain Science, Grossman Center for the Statistics of Mind, Columbia University; Center for Theoretical Neuroscience, Zuckerman Mind Brain Behavior Institute, Kavli Institute for Brain Science, Columbia University

## Abstract

Characterizing interactions between brain areas is a fundamental goal of systems neuroscience. While such analyses are possible when areas are recorded simultaneously, it is rare to observe all combinations of areas of interest within a single animal or recording session. How can we leverage multi-animal datasets to better understand multi-area interactions? Building on recent progress in large-scale, multi-animal models, we introduce **NeuroPaint**, a masked autoencoding approach for inferring the dynamics of unrecorded brain areas. By training across animals with overlapping subsets of recorded areas, NeuroPaint learns to reconstruct activity in missing areas based on shared structure across individuals. We train and evaluate our approach on synthetic data and two multi-animal, multi-area Neuropixels datasets. Our results demonstrate that models trained across animals with partial observations can successfully in-paint the dynamics of unrecorded areas, enabling multi-area analyses that transcend the limitations of any single experiment. Code is available at the following github repository: NeuroPaint

## 1 Introduction

Understanding how brain areas coordinate their activity to support complex behaviors, such as memory-guided actions and perceptual decision-making, is a central challenge in systems neuroscience [17]. Advances in electrophysiological recording techniques now enable single-cell, single-spike resolution measurements across dozens of interconnected brain areas [25, 43, 44]. These developments have driven the collection of brain-wide datasets from hundreds of behaving mice, creating new opportunities to study distributed neural computation at scale [28, 10, 27, 30]. Capitalizing on these datasets requires computational methods capable of modeling inter-area interactions across animals and tasks.

Traditional approaches to modeling inter-area interactions rely on pairwise neuronal correlations [12] or low-dimensional linear methods such as communication subspace analysis [39]. More expressive models, such as DLAG [15], mDLAG [16], and dCSFA [22], capture aspects of temporal and spatial dependencies but typically assume a fixed communication structure and can scale poorly to large datasets. Most recently, large-scale, multi-animal approaches like multi-task-masking (MtM) have shown that pretraining across animals with recordings from overlapping brain areas can improve cross-area prediction [61]. However, these methods are limited to modeling only the areas observed within a given recording session and do not take advantage of a key opportunity in multi-animal datasets: using data from multiple recording sessions across animals to infer activity in unrecorded areas of any given session.

In this paper, we introduce a new framework for analyzing multi-animal, multi-area recordings, NeuroPaint, that explicitly leverages shared anatomical structure across animals to model both recorded and unrecorded brain areas. Building on the masked transformer architecture introduced in [61], our approach incorporates two key innovations: (1) a masking strategy tailored for unrecorded areas, in which unrecorded brain areas are treated the same as masked areas during inference; (2) an architecture that models latent dynamics separately for each brain area, enabling interpretation of area-to-area interactions. To our knowledge, this is the first method specifically designed to infer the dynamics of unrecorded brain areas using data from both other animals and other sessions of the same animal.

We evaluate our method on synthetic data and two large-scale, brain-wide mouse datasets [9, 28]. We find that training across animals with overlapping subsets of recorded brain areas enables NeuroPaint to reliably infer the latent dynamics of missing areas. We compare our approach to LFADS [46] and generalized linear regression, in which we directly predict activity in missing areas from observed activity, demonstrating that our inferred latents provide significantly more predictive information about unrecorded areas. Taken together, this work introduces a new paradigm for analyzing brain-wide activity, demonstrating how shared structure across animals can be exploited to in-paint dynamics from missing brain areas and opening new possibilities for multi-area analyses that transcend the limitations of single-subject recordings.

## 2 Related work

### Multi-area models.

Advances in electrophysiology now enable simultaneous recordings across multiple brain areas [25, 44, 59, 50]. To study concurrent signaling among distributed neuronal populations [1, 24, 28], generative models such as DLAG and mDLAG have been developed to uncover shared latent dynamics across brain areas [22, 15, 16]. While these models offer interpretability, they typically assume fixed bidirectional communication structures and suffer from limited scalability. Multi-area recurrent dynamical models have also been proposed to capture inter-area interactions more flexibly [20, 35, 34, 14]. However, none of these approaches can infer the dynamics of brain areas that are unrecorded during a given session.

### Stitching or quilting multi-session recordings.

Recently, several groups have developed so-called “stitching” [42] or “quilting” [53] approaches to infer functional connectivity or latent dynamics from multi-session recordings with partially overlapping sets of neurons. Some of these methods directly estimate the covariance [5, 6, 63], noise correlations [41, 55], or functional connectivity using generalized linear models [42] and graphical models [53, 7], while others infer latent factors or neural dynamics across sessions [51, 31]. Among these, LFADS [33] is most relevant to our work, as it infers latent dynamics using a nonlinear state space model and can be extended to multi-session settings via linear stitchers. However, state space approaches process time points sequentially and impose strong assumptions on temporal dynamics and noise structure, limiting their scalability and flexibility in large-scale, multi-animal datasets. Moreover, these approaches cannot directly infer neural dynamics in unrecorded brain areas.

### Large-scale models for neural analysis.

Recent work suggests that scaling models across animals and brain areas can be beneficial, as neural activity exhibits shared structure across individuals and regions [37, 2, 58, 60–62]. Transformer-based models have emerged as powerful tools for modeling these multi-animal neural datasets, including methods such as POYO+ [3], a multi-task decoder across animals; NDT [58], which uses masked modeling for neural prediction; and MtM [61] and NEDS [62], which utilize multi-task-masking to learn spatiotemporal structure in neural activity and the bidirectional relationship between neural activity and behavior. However, these models overlook a key opportunity in multi-animal, multi-area datasets: inferring neural dynamics in unrecorded brain areas of one animal by leveraging shared structure across animals.

## 3 Methods

**Figure 1:**
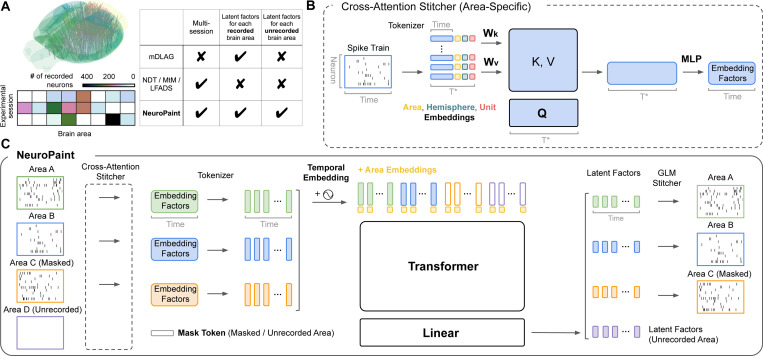
Schematic illustration of NeuroPaint. **(A)** Neuropixels probes only record a subset of brain areas simultaneously. The brain schematic summarizes probe locations across IBL sessions, and the table below shows variability in recorded neuron counts by area and session. In contrast to previous approaches (e.g., mDLAG [16], NDT [57, 58], MtM [61], LFADS [33]), NeuroPaint is the first method that can infer latent dynamics for each brain area, including unrecorded areas, using large-scale multi-session, multi-animal datasets. **(B)** We use a cross-attention stitcher to convert spike data into tokens, and concatenate area, hemisphere, and unit embeddings to form the keys and values. We use learnable latent tokens as queries to reduce input length and produce area-specific embedding factors. Bold symbols indicate parameters shared across sessions, while non-bold symbols denote area-specific parameters. **(C)** NeuroPaint uses a transformer-based architecture with cross-attention stitchers (as shown in B) to encode spike counts into area-specific embedding factors that are then tokenized into temporal tokens. We add temporal and area embeddings to the tokens, which are passed through the transformer and a linear layer to produce area-specific latent factors. During training, we mask tokens from sampled brain areas, and predict them using area-specific GLM stitchers.

In this work, we present **NeuroPaint**, a transformer-based masked modeling approach that predicts neural dynamics in both recorded and unrecorded brain areas using observed activity. In multi-animal extracellular recordings, each session samples neurons from only a subset of brain areas, leaving others unrecorded (Fig. 1A, 3A, E). With sufficient overlap across animals, shared structure can be leveraged to infer missing activity. To model this, we randomly mask recorded areas and train the model to reconstruct them, using a set of low-dimensional latents for each brain area (even when unrecorded), to enable inference of missing brain area dynamics across sessions.

### 3.1 Architecture

The architecture of NeuroPaint has four main components (Fig. 1C): (1) a cross-attention [23] stitcher that maps neural activity from each unmasked brain area to area-specific embedding factors; (2) a tokenizer that transforms these embedding factors into tokens and adds mask tokens for masked and unrecorded areas; (3) a transformer encoder that processes all tokens to produce latent factors for each brain area; and (4) a generalized linear stitcher that reconstructs neural activity from latent factors via a linear readout followed by an exponential nonlinearity. Most parameters are shared across sessions, with a few session-specific parameters noted below.

#### Cross-attention read-in stitcher.

We use a cross-attention stitcher (shown in Fig. 1B) to map neural activity into a shared latent space, producing area-specific “embedding factors” that are consistent across sessions. This module builds on the Perceiver-IO architecture [23, 2], using latent tokens to reduce input length. Neural activity is tokenized at the neuron level, with each neuron providing keys and values. A fixed set of learnable latent tokens act as queries, ensuring that the output embedding factors have the same size as the latent tokens. To construct each neuron token, we concatenate the neural activity embedding with three learnable embeddings: an area embedding (encoding the neuron’s brain area), a hemisphere embedding (indicating left or right hemisphere), and a unit embedding (unique to each neuron). The cross-attention stitcher then outputs a distinct set of embedding factors for each brain area. For a more detailed description of the cross-attention stitcher, see Appendix A.2.

We adopt a cross-attention stitcher [2, 3] in place of a linear stitcher [33, 61, 57], as the input-side transformation must be highly expressive. Neural activity can vary substantially across sessions, and aligning it into consistent across-session dynamics requires non-linear transformations. At the same time, minimizing the number of parameters is critical to avoid overfitting. The cross-attention stitcher supports parameter sharing across sessions and brain areas, improving generalization. Only the unit embeddings remain session-specific, as each session includes a distinct set of recorded neurons.

#### Tokenizer for embedding factors.

The cross-attention stitcher produces area-specific embedding factors, which are then tokenized by treating each time step as a separate token. We replace tokens corresponding to masked areas with a learnable mask token. For each unrecorded area, we also include learnable mask tokens, which allows the model to infer latent factors for unrecorded areas at test time. Inspired by masked autoencoders, which use positional embeddings to preserve the spatial location of image patches [21], we add a brain-area embedding to each token to encode its anatomical location. Although area information is already added in the cross-attention stitcher, adding it to mask tokens is crucial to ensure that the model can distinguish which brain area each token represents. Temporal information is encoded using rotary positional embeddings (RoPE) [45, 2].

#### Transformer encoder.

To process the sequence of tokens from multiple areas and animals, we utilize an encoder-only transformer architecture composed of standard transformer blocks followed by a linear layer [52]. By computing interactions between all pairs of tokens in the input sequence, the self-attention mechanism of the transformer encoder allows the model to capture dependencies across brain areas and time steps. The encoder outputs latent factors for all brain areas of interest, including those that were unrecorded in a given session.

#### Generalized linear read-out stitcher.

A generalized linear read-out stitcher maps the latent factors back to neural activity using a linear layer followed by an exponential activation. This component is kept intentionally simple to preserve the interpretability of latent factors, ensuring that those for unrecorded areas closely reflect actual neural activity. Its parameters are specific to each session and brain area. The number of latent factors per area is chosen based on the maximum linear dimensionality of that area’s smoothed neural activity across sessions (see Appendix A.5).

### 3.2 Masking scheme

To enable the model to infer missing activity from unrecorded areas, we utilize an inter-area masking scheme: in each training batch, we randomly select a masking percentage between 0% and 60%, and mask out that proportion of the recorded brain areas. We then train the model to reconstruct their activity. Since our goal is to predict activity in unrecorded brain areas, inter-area masking alone is insufficient for generalization. To address this, we introduce additional loss terms designed to improve the model’s ability to infer activity in unrecorded areas.

### 3.3 Loss function

The loss function consists of three components: the reconstruction loss, consistency loss, and regularization loss. The **reconstruction loss** is a standard Poisson negative log-likelihood and is used to evaluate the accuracy of predicted firing rates against observed spike counts. The **consistency loss** constrains each brain area’s embedding factors to preserve a stable correlation structure between factors across sessions by penalizing deviations from a session-averaged target correlation (see Appendix A.3 and A.4 for details). This constraint helps the model generalize to unrecorded areas in a given session by encouraging the embedding factors to encode information predictive of all observed areas across sessions. Importantly, the consistency loss is applied to embedding factors (see Fig. 1C for definition), which are related to the neural activity through learned nonlinear transformations. That is, we do not assume stable correlation between areas at the level of raw spike data or latent factors. Finally, the **regularization loss** penalizes rapid temporal fluctuations in the latent space, promoting smoother latent dynamics. Formal definitions of the consistency and regularization losses can be found in Appendix A.3.

## 4 Experiments

Our approach builds upon two core assumptions: (1) neural activity in each brain area lies on a ***low-dimensional*** manifold, allowing a fixed set of low-dimensional latent factors to explain most of the variance in the high-dimensional neural data; and (2) a ***consistent and potentially nonlinear mapping*** exists between the latent dynamics of different brain areas. To test our proposed model, we first apply it to a synthetic dataset constructed to satisfy both assumptions. We then evaluate its performance on two large-scale Neuropixels datasets spanning multiple animals and brain areas, where these assumptions are expected to hold approximately.

In the synthetic data, ground truth firing rates from unrecorded areas are available. For the real data, *we treat one recorded area per session as unrecorded* by holding it out during training, allowing evaluation during test with ground-truth data. We measure how well the inferred latent factors capture dynamics in the held-out area by training, during the test phase, a generalized linear model (GLM) to predict firing rates for that area from its inferred factors. Note that these GLMs cannot be learned during training for the held-out areas because their spike data are not made available during training.

All trials used for evaluating the latent factors come from a test set that was excluded during NeuroPaint training. We split this test set into 60% training and 40% testing trials for cross-validation of the GLM performance. Performance is quantified using deviance fraction explained (DFE), a normalized goodness-of-fit metric (see Appendix A.7). We use DFE as an intuitive, bounded metric where 1 indicates perfect prediction, 0 matches a null model using the average firing rate, and negative values indicate worse-than-null performance. However, when the null model closely matches the ground truth, DFE can be low even if the model performs well. Conversely, DFE values near 1 may reflect overfitting—not in the conventional sense of poor generalization across data splits, but in that the model may capture high-frequency spike noise rather than meaningful structure in the firing rates.

### 4.1 Datasets

#### Synthetic dataset.

To generate synthetic neural data, we simulate an autonomous recurrent neural network (RNN) comprising five brain areas, each with 200 RNN units (see Appendix A.6). Units within each area are densely connected (all-to-all), while inter-area connections are sparse (1%). The recurrent weights induce chaotic dynamics, resulting in trial-to-trial variability driven by different initial conditions. Counterintuitively, the chaotic dynamics generated by such randomly connected networks are often low-dimensional—occupying a subspace whose dimensionality is much smaller than the number of RNN units [11]. For each synthetic session, spike trains are produced using a session-specific GLM with sparse weights (2%), mapping RNN activity to spikes. This setup yields distinct neuron populations per session, each arising from partially overlapping subsets of RNN units. In each session, 3 ~ 4 out of 5 areas are designated as “recorded,” and the rest as unrecorded. Each area contains 20 ~ 60 simulated neurons per session.

#### IBL dataset.

We use the International Brain Laboratory (IBL) brain-wide map dataset [28] (Fig. 3A, B). This dataset consists of Neuropixels recordings collected from 12 labs which utilize a standardized experimental pipeline. The recordings target 279 brain areas across 139 adult mice performing the same visual decision-making task. The probe was localized after the experiments using reconstructed histology and the brain areas were annotated. We utilize trial-aligned, spike-sorted data from 20 mice (1 session per mice), and select 8 brain areas for analysis: PO, LP, DG, CA1, VISa, VPM, APN, and MRN (see Appendix A.1 for full area names). From these recordings, we have a total of 21568 neurons for training and evaluation. We bin the spike trains using 10 ms windows and we fix the trial-length to 2 seconds (200 time bins). Trials are categorized into two types: left choice and right choice. Each of the 20 selected sessions has 3 to 7 brain areas simultaneously recorded. We randomly hold out one recorded brain area per session for evaluation.

#### MAP dataset.

The Mesoscale Activity Project (MAP) dataset records neural activity underlying memory-guided movement in mice using Neuropixels probes targeting motor cortex, thalamus, midbrain, and hindbrain regions, spanning 293 cortical and subcortical structures [10] (Fig. 3E, F). It includes 173 sessions from 28 mice. We use trial-aligned, spike-sorted data from 40 sessions across 16 mice, binned in 10 ms windows over fixed 4-second trials (400 time bins). Analyses are restricted to two trial types, *hit left* and *hit right*, which are also used to compute the consistency loss. For our analysis, we focus on 8 brain areas, 6 highly relevant to the task [19, 29, 18, 49, 9] and 2 orbital areas: ALM, lOrb, vlOrb, Pallidum, Striatum, VAL-VM, MRN, and SC (see Appendix A.1 for full area names). Each of the selected sessions has 4 to 6 brain areas simultaneously recorded. We randomly hold out one recorded brain area per session for evaluation.

### 4.2 Baselines

We compare NeuroPaint against linear and non-linear baselines. We use a standard generalized linear model (GLM) for our linear baseline. For our non-linear baseline, we compare to Latent Factor Analysis via Dynamical Systems (LFADS) [33, 46], a sequential variational autoencoder that has demonstrated strong performance in capturing latent neural dynamics. We utilize a re-implemented version of LFADS [38] for all our analyses, as discussed below.

#### GLM.

We implement GLMs that map from instantaneous spike activity in all the recorded areas to instantaneous spike activity in an unrecorded (in the synthetic dataset) or held-out (in the Neuropixels datasets) area. The GLM consists of a linear layer followed by an exponential nonlinearity, and is trained using the Poisson negative log-likelihood loss. We fit a separate GLM for each session and each held-out area.

#### LFADS.

We re-implement multi-session LFADS [46, 33] to model shared neural dynamics across multiple animals. The objective of LFADS is to maximize the likelihood of observed neural activity by reconstructing spike trains from a low-dimensional latent dynamical system. For each session’s neural population, multi-session LFADS learns a linear projection layer (read-in stitcher) to embed the neural activity into a shared latent space. It also learns a corresponding set of read-out stitchers to map the latent representations back to neural activity space. Unlike NeuroPaint, LFADS embeds activity from all recorded areas into a shared latent space but cannot infer latent dynamics for unrecorded areas, as it is only trained to reconstruct observed data and does not use masked modeling to predict missing areas. Most importantly, LFADS lacks area-specific latent factors, which limits its interpretability in multi-area regimes. We apply principal component regression to select the latent dimensionality and to pre-condition both the read-in and read-out stitchers [33] (see Appendix A.5). Further details on architecture, weight initialization, and implementation can be found in Appendix A.10 and A.11.

In summary, we evaluate the ability of each approach to predict neural activity in unrecorded areas, by using a supervised GLM (trained during the test phase) to predict neural activity in held-out areas. Specifically, we compare the predictive power of three types of inputs to the GLM: (1) spike activity from recorded areas (corresponding to the GLM baseline), (2) LFADS latent factors shared across recorded areas, and (3) NeuroPaint’s area-specific latent factors for the unrecorded area. Although we are comparing NeuroPaint’s performance with these other baselines, they are not comparable in the important sense that NeuroPaint is the only approach that explicitly learns separate latent factors for each brain area, including those unrecorded ones, whereas the two baselines cannot infer area-specific latent dynamics.

## 5 Results

### 5.1 Synthetic dataset

**Figure 2:**
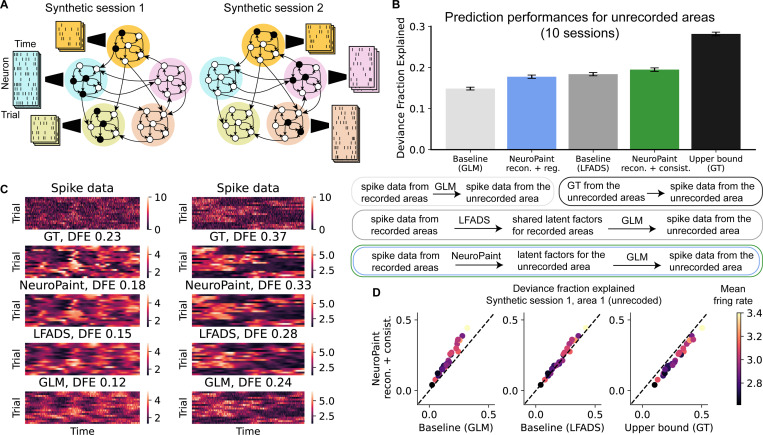
Quantitative and qualitative comparison of NeuroPaint with baseline models on synthetic datasets. **(A)** Synthetic spike trains are generated across 10 sessions using a shared underlying RNN, with five brain areas of 200 units each. Spike trains are generated using session- and area-specific GLMs with sparse, random weights, allowing partially overlapping RNN units to contribute differently across sessions. **(B)** Prediction performance for synthetic neurons in unrecorded areas across 10 sessions. The bar plot displays the mean DFE and one standard error across neurons for each method, while the flow diagram shows the computation of DFE. **(C)** Spike data and ground truth firing rates (GT) for two example synthetic neurons, compared with firing rate predictions from NeuroPaint (trained with reconstruction and consistency losses) and the two baselines (GLM and LFADS). **(D)** Prediction performance for synthetic neurons from area 1 (unrecorded) in session 1. Each dot represents a neuron, showing DFE achieved by NeuroPaint (trained with reconstruction and consistency loss), the two baselines, and the upper bound. Dots are color-coded by each neuron’s mean firing rate.

We evaluate NeuroPaint on a synthetic dataset with known ground truth firing rates, enabling computation of an upper-bound DFE using spike data in the unrecorded areas and true rates. The model is trained on recorded areas from 10 synthetic sessions and evaluated on its ability to infer single-neuron firing rates in unrecorded areas. The architecture follows Section 3.1, excluding hemisphere embeddings due to the absence of hemispheric structure in the simulation. We compare two model variants, one trained with reconstruction plus regularization loss, the other with reconstruction plus consistency loss; combining all three losses (not shown) results in over-regularization and performance degradation below baseline. This is likely due to the fast, high-firing-rate dynamics in the synthetic data; as suggested by an additional experiment on a separate low-firing-rate synthetic dataset, where the full model (all three losses) outperforms the ablated variant lacking one loss term (see Appendix A.6 and Supplementary Fig. 2). We compare each model against GLM and LFADS baselines and the upper bound. The consistency-loss variant outperforms the two baselines and the regularized model, and is the closest to the upper bound (Fig. 2B, D). As shown in Fig. 2C, NeuroPaint more accurately captures temporal structure than GLM, which tends to overfit to Poisson noise, and slightly outperforms LFADS in recovering fine-grained features of the ground truth firing rates. These results show that under the idealized conditions of the synthetic data, *i.e.* low-dimensional activity and consistent cross-area mapping, NeuroPaint can accurately infer neural dynamics in unrecorded areas.

### 5.2 IBL and MAP datasets

In the Neuropixels datasets, although we have ground truth spiking data, the absence of ground truth firing rates precludes computation of an upper-bound DFE. While absolute DFE values may appear lower than those observed in the synthetic data, they reflect the intrinsic performance ceiling set by the low and sparsely modulated firing rates typical of real neuronal activity. Rather than indicating suboptimal performance, these values underscore the challenge of the task and the robustness of the model’s performance under realistic biological constraints.

We train two separate NeuroPaint models, one for the 20 IBL sessions and one for the 40 MAP sessions. In both datasets, we compare NeuroPaint to LFADS, which is the highest performing baseline for single-neuron prediction accuracy in held-out areas. The GLM baseline performs poorly across both datasets due to its inability to model non-linear, cross-area interactions in real neural data [56, 54], and is therefore omitted from the comparison (results are provided in Appendix A.9).

**Figure 3:**
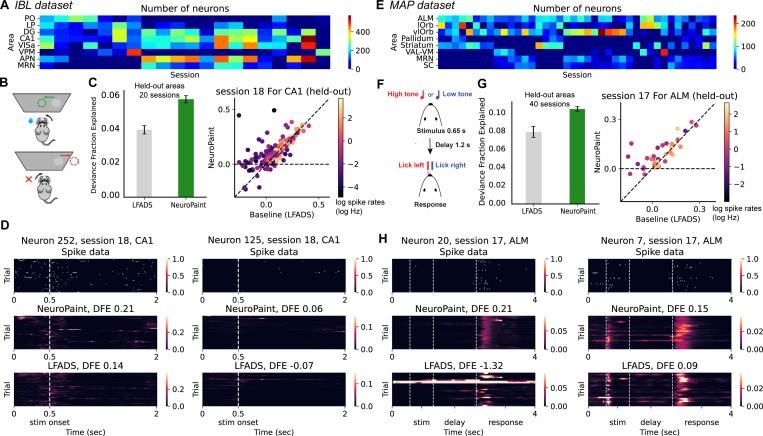
Quantitative and qualitative comparison of NeuroPaint with baseline models on IBL and MAP datasets. **(A)** Number of neurons recorded per area in 20 IBL sessions. **(B)** In the IBL experimental setup, mice perform a visual decision-making task by turning a wheel to the left or right to indicate whether the stimulus is presented on the left or right screen [28]. **(C)** Left: The mean and standard error of DFE for LFADS and NeuroPaint, calculated for neurons from held-out areas pooled across 20 IBL sessions, excluding the worst 1% of neurons for visualization; see Fig. S1A for results without exclusion. Right: Per-neuron DFE for the held-out area (CA1) of session 18. **(D)** Spike trains and predicted firing rates for two example neurons, compared between NeuroPaint and LFADS predictions. **(E)** Number of neurons recorded per area in 40 sessions from MAP dataset. **(F)** The MAP dataset records neural activity from mice performing a memory-guided directional licking task, in which they are instructed to lick left or right according to auditory tones presented before a fixed delay period. [9]. **(G, H)** show similar content to **(C, D)**, but with neurons from held-out areas pooled across 40 MAP sessions (see Fig. S1B for results with the worst 1% exclusion).

Across held-out areas, NeuroPaint outperforms LFADS by a large margin in both the IBL and MAP datasets (Fig. 3C, G; Fig. S1). Example raster plots of individual neurons further show that NeuroPaint captures structured, single-trial dynamics that LFADS fails to recover (Fig. 3D, H). In particular, LFADS exhibits large outlier errors for some neurons (e.g., neuron 20 in Fig. 3H), whereas NeuroPaint delivers more stable and accurate firing rate predictions. In the neural data, which has relatively low firing rates compared to the synthetic data of Fig. 2, the combination of all three loss terms performs best: ablation experiments show that removing either the regularization or the consistency loss degrades performance relative to the full NeuroPaint model (see Appendix A.13, Supplementary Fig. 1). Moreover, increasing the number of parameters in the LFADS model does not close the performance gap to NeuroPaint (see Appendix A.15), suggesting that NeuroPaint’s advantage is not explained by model size alone. These results demonstrate that NeuroPaint not only infers interpretable, area-specific latent factors for unrecorded brain regions, but also achieves state-of-the-art predictive performance.

### 5.3 Interpretable area-specific latent dynamics revealed by NeuroPaint

We use the MAP dataset to illustrate that NeuroPaint generates consistent and interpretable area-specific latent dynamics across trials and sessions. See similar results for IBL dataset in Appendix A.14.

#### Preprocessing.

**Figure 4:**
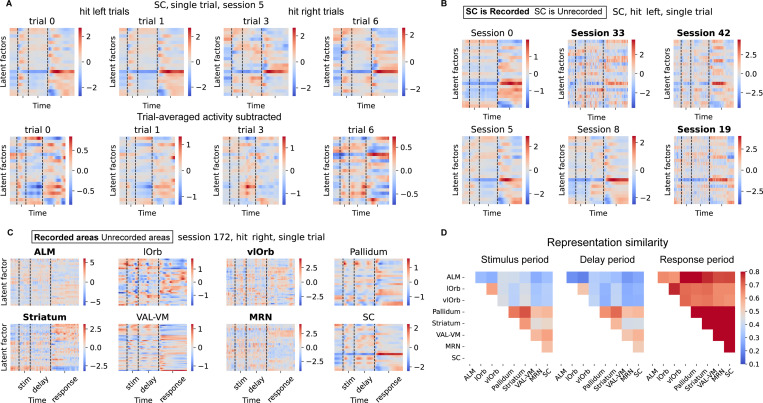
NeuroPaint’s area-specific latent factors are consistent, capture context-dependent variability, and enable inference of area-to-area interactions. **(A)** Inferred latent factors for the Superior Colliculus (SC), an unrecorded area, during example trials across the *hit left* and *hit right* conditions in a single session. The bottom row shows the same latent factors with the trial-averaged activity subtracted. **(B)** Inferred single-trial latent factors for SC (both recorded and unrecorded) across six sessions. **(C)** Latent factors for all eight areas (both recorded and unrecorded) in a *hit right* trial from a single session in the MAP dataset. **(D)** Representation similarity analysis (RSA) of latent factors across eight brain areas (recorded and unrecorded) for the stimulus, delay, and response periods in the MAP dataset, with values averaged across trials and pooled over sessions.

To highlight dynamic structure over static offsets, we subtract the temporal mean of each trial from each latent factor before computing correlations or visualizing dynamics in Fig. 4. This preprocessing step helps mitigate spurious correlations driven by differences in the latent factor’s non-zero temporal means.

#### Consistent and context-dependent latent dynamics.

We evaluate (1) the consistency of latent factors across trials for both recorded and unrecorded areas, and (2) the context-dependent variability of latent factors that reflects distinct behavioral conditions, such as *hit left* vs. *hit right* trials where mice respond by licking in different directions. We find that the inferred area-specific latent factors exhibit consistent temporal structure across trials within the same behavioral context (Fig. 4A), even in sessions where the corresponding area is unrecorded (Fig. 4B). Additionally, the latent factors exhibit clear context-dependent variability that captures task-relevant differences; for instance, the latent factors for area SC show distinct patterns between hit left and hit right trials (Fig. 4A). To quantify the consistency and context-dependent variability, we compute the Pearson correlation between latent factors across all trial pairs, averaged over brain areas (see Appendix Table 5). First, latent factors from the same brain areas exhibit positive correlations across trials, regardless of whether those areas were recorded or not in a given session. Second, trial pairs of the same type have higher correlations than those of different types, suggesting that the latent factors capture context-dependent variability. (see Appendix A.16 for more details)

To directly evaluate whether the latent factors capture stimulus- or behavior-dependent variability, we performed decoding analysis from the latent factors. We found that the latent factors in most areas reliably support decoding for both stimulus and behavioral choice, demonstrating that they encode meaningful, task-relevant information. (see Appendix A.17 for more details)

#### Inferring area-to-area interactions from inpainted neural data.

We demonstrate that NeuroPaint’s inferred latent factors enable novel analyses of area-to-area interactions during behavior. As shown in Fig. 4C, the latent factors from both recorded and unrecorded areas in a single trial reveal rich and distinct temporally structured activity across the 8 selected brain areas. With a fully inpainted neural picture at a single-trial resolution across 40 sessions, NeuroPaint enables analyses on area-to-area interactions that were previously infeasible with Neuropixels recordings, which lack simultaneous coverage of all areas. As one such analysis, we quantify representational similarity across all pairs of brain areas. For each area and each trial period (stimulus, delay, and response), we first compute a time-by-time representational dissimilarity matrix (RDM), defined as one minus the pairwise correlation between flattened latent factors. We then measure area-to-area similarity by correlating the upper-triangular elements of these RDMs, averaged across trials over multiple sessions (Fig. 4D). This analysis reveals that inter-area relationships evolve across behavioral epochs: similarity is relatively low during stimulus and delay periods but increases during the response period, which is consistent with previous findings of increased inter-area coordination during motor output [9]. While Chen et al. [9] were limited to analyzing interactions between ALM and a few simultaneously recorded areas, NeuroPaint overcomes this limitation by enabling comparisons across all area pairs, even including unrecorded areas, via latent inpainting. Furthermore, we observe that the two orbital areas (lOrb and vlOrb) exhibit high mutual similarity, reflecting their shared anatomical and functional roles [32]. In contrast, these areas show weak similarity to the remaining brain areas, even during the response period, which aligns with prior knowledge about the involvement of the selected non-orbital areas in task-relevant computations [19, 29, 18, 49, 9].

## 6 Discussion

In this work, we introduce NeuroPaint, a masked transformer-based model that infers the dynamics of unrecorded brain areas by leveraging shared activity structure across animals. Our experiments on synthetic and large-scale Neuropixels datasets show that NeuroPaint outperforms both linear and non-linear baselines, including LFADS and GLMs, in predicting single-neuron activity in held-out areas. Beyond predictive accuracy, NeuroPaint produces interpretable latent dynamics that are consistent across sessions and sensitive to behavioral context, enabling new forms of cross-area and cross-session analysis that were inaccessible with existing tools.

Despite these strengths, several limitations remain. First, training NeuroPaint is computationally expensive, as the self-attention mechanism scales quadratically with the number of brain areas and time steps. Future work could address this by incorporating sparse or low-rank attention mechanisms [8]. Second, the latent dimensionality for each brain area is not manually selected, it is estimated via a principled procedure (see Appendix A.5). Nonetheless, the procedure involved design choices that introduce some arbitrariness, and we find that the selected number of latent factors often exceeds the intrinsic dimensionality of the predicted firing rates in each brain area (see Appendix A.18). More automated hyperpameter selection methods, such as Population Based Training [26], could reduce this arbitrariness. Finally, while we demonstrate proof-of-concept results on two datasets, our experiments involve a limited subset of brain areas and sessions relative to the full datasets. Scaling to hundreds of sessions is straightforward, but extending to hundreds of brain areas will require architectural innovations and improved training strategies. Nonetheless, this represents a critical step toward building truly brain-wide models.

## Supplementary Material

Supplement 1
