# Supplementary material for: Inpainting the Neural Picture: Inferring Unrecorded Brain Area Dynamics from Multi-Animal Datasets
Source: ArXiv. 2025 Oct 13:arXiv:2510.11924v1. Preprint. [Version 1] (PMC12633159)
Supplement: Supplement 1 [file NIHPP2510.11924v1-supplement-1.pdf]

A Technical Appendices and Supplementary Material

A.1 Dataset details

This section provides the full anatomical names and corresponding acronyms of the brain areas analyzed throughout the paper.

IBL dataset. The posterior thalamus (PO), lateral posterior nucleus (LP), dentate gyrus (DG), hippocampal CA1 (CA1), anterior/anteromedial visual area (VISa), ventral posteromedial nucleus (VPM), anterior pretectal nucleus (APN), and midbrain reticular nucleus (MRN).

MAP dataset. The anterior lateral motor cortex (ALM), orbital area, lateral part (lOrb), orbital area, ventrolateral part (vlOrb), pallidum combined with globus pallidus, external segment (Pallidum), striatum combined with caudoputamen (Striatum), ventral anterior-lateral complex of the thalamus combined with ventral medial nucleus of the thalamus (VAL-VM), midbrain reticular nucleus (MRN), and superior colliculus, motor related (SC). ALM is identified following methods described in [47].

A.2 Generative process

For the N_r neurons in area r, we construct neural data tokens $Z_{X_r} \in \mathbb{R}^{N_r \times T^{*'}}$ by concatenating neural acivity $X_r \in \mathbb{R}^{N_r \times T}$ with area, hemisphere, and unit embeddings: $Z_{\text{area}} \in \mathbb{R}^{D_a}, Z_{\text{hemi}} \in \mathbb{R}^{D_h}, Z_{\text{unit}} \in \mathbb{R}^{D_u}$, where $T^{*'} = T + D_a + D_h + D_u$. We then compute keys ($K_r$) and values ($V_r$) for the cross-attention module using linear projections $W_K, W_V \in \mathbb{R}^{T^* \times T^{*'}}$. A shared set of learnable latent tokens $Z_0 \in \mathbb{R}^{P \times T^*}$ acts as the query Q. The resulting cross-attention output is projected via a multi-layer perceptron to obtain embedding factors $Z_{\text{embed},r} \in \mathbb{R}^{P \times T}$.

For each trial, a masking proportion p is chosen from a uniform distribution from 0 to 0.6. If $p <= 0.05$, then no areas are masked, otherwise, if the trial has recordings from R areas (where held-out areas are considered unrecorded), then Ceiling[$p * R$] areas are randomly chosen to be masked. A variable M_r is set to 1 for masked areas and 0 for unmasked areas. The embedding factors $Z_{\text{embed},r}$ are tokenized by treating each timestep as a token, resulting in T tokens $Z_r^{\text{token}} \in \mathbb{R}^{T \times H}$, where an MLP projects each token from $\mathbb{R}^P$ to $\mathbb{R}^H$. For masked or unrecorded areas, these neural data tokens are replaced by a learned mask token Z_{mask}. We define a binary indicator $U_r \in \{0, 1\}$ to denote whether area r is unrecorded. Each token is then concatenated with a new learnable area embedding $Z'_{\text{area}} \in \mathbb{R}^{D_{a'}}$, producing the input tokens $Z_r^{\text{input}} \in \mathbb{R}^{T \times H'}$, $H' = H + D_{a'}$. These token sequences are passed through a transformer (with RoPE applied to queries and keys), and the outputs (also $\in \mathbb{R}^{T \times H'}$) are projected via $W_r^{\text{latent}} \in \mathbb{R}^{H' \times D_r}$ to obtain latent factors $Z_{\text{latent},r} \in \mathbb{R}^{T \times D_r}$. Finally, firing rate predictions $\hat{X}_r$ are computed via a linear readout $W_r^{\text{rate}} \in \mathbb{R}^{D_r \times N_r}$ followed by exponentiation.

The training procedure is as follows:

$$
\begin{array}{ll|ll}
\textbf{Cross-attention stitcher} & & \textbf{Remaining modules of NeuroPaint} & \\
Z_{X_r} & = [X_r, Z_{\text{area}}, Z_{\text{hemi}}, Z_{\text{unit}}] & Z_r^{\text{token}} & = \text{Tokenizer}(Z_{\text{embed},r}) \\
K_r, V_r & = W_K(Z_{X_r}^T), W_V(Z_{X_r}^T) & \delta_r & = \begin{cases} 1 & \text{if } M_r = 0 \wedge U_r = 0 \\ 0 & \text{otherwise} \end{cases} \\
Q & = Z_0 & Z_r^{\text{input}}(t) & = [(1 - \delta_r) Z_{\text{mask}} + \delta_r Z_r^{\text{token}}(t),\ Z'_{\text{area}}] \\
Z_{\text{cross-attn},r} & = \text{Cross-Attention}(Q, K_r, V_r) & Z_{\text{latent},r} & = (\text{Transformer}(\text{RoPE}(Z_r^{\text{input}})))W_r^{\text{latent}} \\
Z_{\text{embed},r} & = \text{MLP}(Z_{\text{cross-attn},r}) & \hat{X}_r^T & = \text{Poisson}(\exp(Z_{\text{latent},r} W_r^{\text{rate}}))
\end{array} \tag{1}
$$

The model assumes a Poisson emission process with time-varying rates. For notational simplicity, we describe the generative model using data from one trial in one session. Symbols with the subscript r denote area-specific parameters, while those without r are shared across areas. Most parameters are shared across sessions, except for Z_{unit} and W_r^{rate}.

A.3 Loss function details

The consistency and regularization losses are formally defined below.

To ensure the embedding factors maintain a stable correlation structure across sessions and penalize deviations from a session-averaged target, we introduce the following consistency loss.

Definition A.1 (**Consistency loss**). *Let $Z^{(b)}_{embed,r}(t) \in \mathbb{R}^P$ denote the embedding factors for area $r \in [R]$ at time t under trial type $b \in [B]$. Define $K^{(b)}_{model}(r,r') \in \mathbb{R}^{P\times P}$ as the Pearson correlation matrix between $Z^{(b)}_{embed,r}(t)$ and $Z^{(b)}_{embed,r'}(t)$ computed across time after aggregating time steps over trials of type b within a batch. The corresponding target correlation matrix $K^{(b)}_{target}(r,r') \in \mathbb{R}^{P\times P}$ is obtained by averaging the correlation between $Z^{(b)}_{embed,r}(t)$ and $Z^{(b)}_{embed,r'}(t)$ across time steps in trials of type b, aggregated over sessions. The consistency loss is then defined as follows.*

$$\mathcal{L}_{\text{consist.}} = \sum_{b}^{B} \sum_{r'=1}^{R} \sum_{r=1}^{R} 1 - \cos\left(\text{vec}\left(K^{(b)}_{\text{target}}(r,r')\right), \text{vec}\left(K^{(b)}_{\text{model}}(r,r')\right)\right), \tag{2}$$

where $\cos(\cdot,\cdot)$ denotes the cosine similarity, and vec$(\cdot)$ is a vectorization operator that flattens the correlation matrix, using only the upper triangular elements when $r = r'$.

The consistency loss encourages each area's embedding factors to encode information predictive of all areas observed across sessions, in addition to those recorded in the current session, thereby improving generalization to unrecorded areas. In practice, we approximate the session-averaged target $K^{(b)}_{\text{target}}(r,r')$ using a buffer of past batch correlations, computed with an exponential moving average (EMA) of the cross-attention stitcher to improve stability (Appendix A.4) [48].

Under the assumption that neural dynamics evolve smoothly over time, we define the following regularization loss.

Definition A.2 (**Regularization loss**). *Let $Z_{latent,r}(t) \in \mathbb{R}^{D_r}$ denote the latent factors at time t for area $r \in [R]$ and let $Z_{latent,r}(t,i)$ denote its i-th component. The regularization loss is defined as follows.*

$$\mathcal{L}_{\text{reg.}} = \sum_{t=1}^{T-1} \sum_{r=1}^{R} \sum_{i=1}^{D_r} |\, Z_{\text{latent},r}(t+1,i) - Z_{\text{latent},r}(t,i)\,|\,. \tag{3}$$

The regularization loss penalizes abrupt changes across consecutive time steps in the latent space, thereby improving temporal smoothness of the learned latent factors.

The relative weighting of the loss terms was selected to ensure that the reconstruction loss remains the primary driver during training, while the consistency and regularization terms serve as auxiliary constraints. Specifically:

- The reconstruction loss is scaled by a weight of 1 and normalized by the number of timesteps and neurons.
- The consistency loss is scaled by a weight of 1 and normalized by the number of area pairs.
- The regularization loss is scaled by a weight of 0.1 and normalized by the number of timesteps and latent factors.

A.4 Exponential moving average (EMA) of the cross-attention stitcher

To stabilize the correlation target calculated from the area-specific embedding factors, we maintain an EMA version of the cross-attention stitcher during training. At each iteration, the parameters in the EMA version are updated as a weighted sum of their previous values and the current weight of the cross-attention stitcher in the main model, controlled by a decay rate α. Specifically, the EMA parameter θ is updated as follows:

$$\theta^{EMA} \leftarrow \alpha\theta^{EMA} + (1-\alpha)\theta \tag{4}$$

To reduce bias in early training when parameter estimates are still unstable, we adapt the decay rate α based on the number of iteration steps n_{step}:

$$\alpha = \min(1 - \frac{1}{n_{\text{step}}+1}, \alpha_{\max}) \tag{5}$$

which increase α as training progresses. Here $\alpha_{\text{max}} = 0.999$. This ensures faster adaptation in the early iterations and more stable averaging later on. We use the EMA cross-attention stitcher to compute the area-specific embedding factors and then compute the correlation between embedding factors for each batch, and store them in a running buffer. These stored correlation matrices are then averaged to generate the target correlation matrix for each area pair and trial type for the current iteration.

A.5 Determining the area-specific latent factor dimension in NeuroPaint and LFADS

NeuroPaint. To determine the number of latent factors for each brain area, we estimated the dimensionality of smoothed neural activity across sessions. For each area and session, we first smoothed the spike trains with a Gaussian window (standard deviation = 50 ms), concatenated all trials, and subtracted the mean to center the data. We then computed the participation ratio of the resulting activity matrix, which provides a measure of its linear dimensionality [13]. This yielded one dimensionality estimate per area per session. To ensure sufficient capacity, we set the number of latent factors for each brain area to the maximum participation ratio observed across sessions for that area, plus a margin of 10 dimensions. We observed that for most brain areas, the estimated dimensionality saturated as the number of recorded neurons increased, suggesting that the low-dimensional assumption we mentioned at the end of the introduction is approximately satisfied by the neural data. This procedure ensures that NeuroPaint is equipped with enough latent factors to model the full range of dynamics expressed in each area.

Area	Dim
PO	61
LP	84
DG	72
CA1	39
VISa	35
VPM	43
APN	58
MRN	24
ALM	29
lOrb	31
vlOrb	37
Pallidum	23
Striatum	31
VAL-VM	49
MRN	22
SC	21

Table 1: NeuroPaint latent factor dimensions picked for areas from IBL and MAP datasets.

LFADS. The multi-session LFADS [33] uses per-session linear read-in and read-out layers to align neural activity from different sessions into a shared latent space. These session-specific "stitching" layers map observed spike counts to input factors (read-in) and latent factors to firing rates (read-out). The shape of these matrices can vary to match the number of neurons recorded in each session. A shared encoder, generator, and factor matrix are shared across sessions and learned jointly from all sessions. The per-session read-in and read-out matrices are learned using data from only the corresponding session. To promote alignment across sessions, we follow the standard procedure to initialize the weights of the read-in and read-out matrices using principal component regression (PCR) [38]. PCR maps the trial-averaged firing rates from each individual session to the shared principal components across all sessions. This provides the model with an input that is already in a shared subspace and is critical for ensuring a shared set of dynamics is learned. We first reshape the trial-averaged firing rates from all sessions into a matrix of size $(n_{\text{timepoints}} \times n_{\text{conditions}}) \times (n_{\text{sessions}} \times n_{\text{neurons}})$, and PCA is applied to identify a set of global principal components (PCs). We find the number of PCs needed to explain 90% of the variance, which defines the dimensionality of the latent factors used in LFADS. Each session's data is then projected onto this global PC space via Ridge regression, and the

resulting weights and biases from the trained regression model are saved to be used to initialize the read-in weights and biases in LFADS.

A.6 Details of the simulated recurrent neural network in the synthetic dataset

To generate synthetic spike data with multi-area dynamics, we simulated a continuous-time recurrent neural network (RNN). This RNN is composed of 5 areas, each area contains 200 units. Each unit has a $\tanh$ nonlinearity and with a time constant $\tau = 25$ ms. We simulate the network using a timestep of $\Delta t = 10$ ms. Unit activity evolves according to the following update rule:

$$h_{t+1} = (1 - \beta)h_t + \beta \tanh(W h_t) \tag{6}$$

Here $\beta = \frac{\Delta t}{\tau}$. W is the recurrent connectivity between units. h_t denotes unit activities at time step t.

The network receives no external input, its activity is driven entirely by autonomous recurrent dynamics. Each recurrent connection has a weight sampled from a normal distribution $W_{ij} \sim \mathcal{N}(0, g^2/N)$, here W_{ij} is the weight between unit i and unit j, $N = 1000$ is the total number of units in the network, we pick $g = 3$ so that we will have chaotic circuit dynamics [40]. Connectivity between areas is sparse: each pair of units are connected with 1% probability. Units within the same area have all-to-all connectivity.

For each trial, we randomly initialize the unit activity in the network. Due to chaotic dynamics, each trial will have qualitatively different dynamics. We simulate trials from the same RNN across synthetic sessions.

For each synthetic session, we simulate neuronal spike trains from the unit activity using GLMs. Each session has its own set of synthetic neurons, with the number of neurons in each area sampled uniformly from the range $[20, 60]$. The number of trials in each synthetic sessions are sampled uniformly from the range $[200, 300]$.

Each area-specific and session-specific GLM first projects the unit activity onto neuronal instantaneous log firing rates using a sparse linear layer (2% sparsity). The log firing rates for each synthetic neuron are then scaled to a fixed range ([0,2]). Next, we pass the log rates to an exponential nonlinearity and a Poisson emission process. For each session, we held-out spike activities generated from 1-2 RNN areas as unrecorded.

In summary, partially overlapped RNN units contribute to the simulated spike trains across synthetic sessions.

A.7 Deviance fraction explained (DFE)

Deviance fraction explained is a metric that ranges from 0 to 1, where a larger value indicates a better model fit. It is defined as:

$$1 - \frac{D_{\text{model}}}{D_{\text{null}}}$$

where the deviance terms are given by:

$$\begin{aligned} D_{\text{model}} &= \log p(\mathbf{x}_{1:T} | M_{\text{sat}}) - \log p(\mathbf{x}_{1:T} | M) \\ D_{\text{null}} &= \log p(\mathbf{x}_{1:T} | M_{\text{sat}}) - \log p(\mathbf{x}_{1:T} | M_{\text{null}}) \end{aligned}$$

Here, M_{sat} represents the **saturated model**, while M_{null} represents the **null model**. Both the saturated and null models assume that the instantaneous spike data is generated from an i.i.d. Poisson distribution. The **saturated model** (M_{sat}) assumes that the mean of the Poisson distribution is given by the instantaneous spike count, while the **null model** (M_{null}) assumes that the mean is given by the time-averaged and trial-averaged spike count.

A.8 Evaluation on Synthetic Data with Lower Firing Rates (Matching Real Data Statistics)

To better align our synthetic data with the firing rate statistics observed in the real neural recordings, we generated an additional synthetic dataset, where the log firing rates of each neuron were scaled to a fixed range of $[-3, 3]$, in contrast to the range $[0, 2]$ used in the previous synthetic dataset shown in Fig. 2. This adjustment results in lower and more realistic firing rates.

We evaluated the performance of several models for predicting neural activity in the unrecorded brain areas on this synthetic dataset: two baselines (GLM and LFADS) and multiple variants of NeuroPaint (including the linear version described in A.12 and other variants trained with different combinations of loss terms). Overall, we found that NeuroPaint trained with all three loss terms (reconstruction, consistency and regularization) achieved the best performance for this low-rate synthetic dataset (see Fig. S2), unlike for the higher-rate synthetic dataset discussed in the main text (Fig. 2). Empirically, we found that including the consistency loss was particularly important for improving NeuroPaint's performance, enabling it to outperform the baselines.

It is important to note that for the synthetic dataset, the GLM baseline also performs very well, suggesting that a linear model is sufficient to predict neural activity in one area based on data from other areas, i.e. to capture inter-areal communication in this simplified setting. In future work, we plan to construct more complex and biologically realistic synthetic datasets that include strong nonlinear inter-areal dependencies, to better evaluate the advantage of nonlinear models.

A.9 GLM baseline performance on the IBL and MAP datasets

We evaluated GLM baselines on both the IBL and MAP datasets. For the IBL dataset, the mean deviance fraction explained (DFE) across neurons in the held-out areas was extremely negative (-3.8×10^{32}), indicating severe overfitting or model mismatch. Notably, 90.7% of neurons had negative DFE values. For the MAP dataset, the mean DFE was -5.1×10^{23}. 63.3% of neurons exhibited negative DFE values.

A.10 Model and hyperparameter details

NeuroPaint. To avoid overfitting, we add dropout (40%) to all the attention layers and a dropout (20%) at the very beginning. Other hyperparameters of the NeuroPaint model is listed in Table 2. Notations for the hyperparameters are introduced in A.2. For synthetic dataset, we use 24 latent factors for each area. For the IBL and MAP dataset, we set the dimensionality of latent factors according to A.5.

Hyperparameter	Value
D_a	20
D_h	3
D_u	50
T	400 (MAP), 200 (IBL)
T^*	512
P	48
H	236
D'_a	20
H'	256
number of transformer layers	5

Table 2: Hyperparameters used for NeuroPaint.

LFADS. We use a version of LFADS (*lfads-torch*) re-implemented in PyTorch by [38]. The hyperparameters of our multi-session LFADS are provided in Table 3. The shared encoder, generator, and factor matrix comprise about 0.3 million parameters. When including session-specific read-in and read-out matrices, the total number of model parameters increases to 0.4 million, 0.5 million, and 0.6 million for the synthetic, IBL and MAP datasets. The latent factor dimension of multi-session LFADS is chosen via PCR (Appendix A.5). We set the latent factor dimension to 28, 11, and 27 for the synthetic, IBL and MAP datasets. The LFADS model computes KL divergence between posteriors and priors for both initial condition and inferred input distributions, which are added to the reconstruction cost in the variational ELBO. The priors are multivariate normal for the initial conditions and autoregressive multivariate normal for the inferred inputs.

NeuroPaint-Linear. The linear, session-specific, and area-specific stitchers take as input the flattened population activity from 3 neighboring bins centered around the target time point. Prior to this, the binned spike counts are smoothed using a 1D Gaussian kernel with a standard deviation of 5

Hyperparameter	Value
Initial Condition Encoder Dimension	100
Controller Input Encoder Dimension	100
Controller Input Lag	1
Controller Dimension	100
Controller Output Dimension	6
Initial Condition Dimension	100
Generator Dimension	100
Controller Output Prior Temporal Decay Constant	10
Controller Output Prior Innovation Variance	0.1
Initial Condition Prior Mean	0
Initial Condition Prior Variance	0.1
Dropout Rate	0.02
Coordinated Dropout Rate	0.3
Weight Decay	0.0
Learning Rate	0.0003
Batch Size	1024

Table 3: Hyperparameters used for multi-session LFADS.

time bins. The stitchers output area-specific embedding factors, which, along with the latent factors, are set to have the same dimensionality as in the original NeuroPaint model. The session-shared, reduced-rank linear transformation applied to the concatenated embeddings has a fixed rank of 20. The population activity in the masked areas, which the model is trained to predict, is also smoothed using a Gaussian kernel with the same standard deviation of 5 time bins.

A.11 Training details

NeuroPaint. We trained our model on 2-12 Nvidia A40 or A100 GPU using AdamW optimizer for 1000 epochs with a learning rate of $(1e^{-3}/256 \times \text{global batch size})$ using a OneCycleLR scheduler. We put a weight decay 0.01 to avoid overfitting. We utilized a batch size of 16 on each GPU during the training. Global batch size is $16\times$ the number of GPU nodes. We split our dataset based on the session to training, validation, and test set with a proportion of 60%, 20%, and 20%. We saved the model checkpoint based on the validation loss. The 10-session model for the synthetic dataset is trained with 2 GPU nodes in around 6 hours. The 20-session model for the IBL dataset is trained with 4 GPU nodes in around 35 hours. The 40-session model for the MAP dataset is trained with 12 GPU nodes in around 20 hours.

LFADS. We train the multi-session LFADS using the AdamW optimizer without a learning rate scheduler, which is the default setting in the *lfads-torch* package. All multi-session LFADS models are trained on a single Nvidia A40 or A100 GPU for 1000 epochs. The best model checkpoint is selected based on the reconstruction performance on the validation set. The 10-session model for the synthetic dataset is trained in under 4 hours. The 20-session LFADS for the IBL dataset takes approximately 10 hours, while the 40-session LFADS for the MAP dataset is trained in about 16 hours.

NeuroPaint-Linear. Training details are provided in Appendix A.12. All models are trained on a single NVIDIA RTX 2080 Ti GPU and complete within one hour.

A.12 NeuroPaint-Linear

We introduce NeuroPaint-Linear, a *linear* variant of the original NeuroPaint model. Designed for simplicity and interpretability.

Architecture NeuroPaint-Linear retains the core architectural components of NeuroPaint, comprising three main components:

1. **Linear, session-specific, and area-specific** stitchers, which map population activity to a set of embedding factors.
2. A **low-rank, session-shared** weight matrix, which transforms the concatenated embedding factors (across all areas) into area-specific latent factors, capturing a mapping between the latent dynamics of different brain areas that remains consistent across sessions and animals.
3. **Linear, session-specific, and area-specific** readout layers, which map latent factors to predicted population activity (for all the recorded and unrecorded areas).

While the overall structure closely mirrors that of NeuroPaint, NeuroPaint-Linear introduces three simplifications:

1. The cross-attention readin-stitcher is replaced by a simple linear mapping.
2. The transformer encoder is replaced with a reduced-rank linear transformation, restricting inter-area mappings to linear operations.
3. In contrast to the nonlinear NeuroPaint, where embeddings for masked or unrecorded areas are replaced with a learned mask token after tokenization, we set these embedding factors to zero directly, bypassing the tokenizer.

In addition, NeuroPaint-Linear operates in a local and time-independent fashion: latent factors and the corresponding predicted activity are computed independently at each time step, using only the recorded activity from a short temporal window (3 time bins, *i.e.* ± 1 bin) around that time—without leveraging long temporal context.

Training details Training follows a similar inter-area masking strategy used in NeuroPaint. For each epoch, one recorded area per session is randomly selected and masked from the input. The model is then trained to predict the activity of the masked area.

Optimization is performed using the L-BFGS algorithm in full-batch mode, minimizing the mean squared error computed across all time steps and all neurons in the masked areas over sessions. For each training epoch, L-BFGS is reinitialized from scratch and run until convergence. We train models for each of the MAP and IBL datasets for 50 epochs each (we only show results for the MAP dataset), using the default L-BFGS hyperparameters provided by PyTorch. We chose L-BFGS and mean squared error due to their stability during training and their effectiveness.

Comparison with standard reduced-rank regression methods NeuroPaint-Linear differs fundamentally from standard reduced-rank regression approaches used in encoding models [4, 36] and inter-areal correlational analyses (e.g., communication subspace) [39], both in its objectives and methodology. The key innovations lie in the introduction of a session-shared mapping from the full set of area-specific embedding factors to area-specific latent factors, and the use of an inter-area masking strategy during training. Together, these components enable NeuroPaint-Linear to predict the dynamics of unrecorded brain areas based solely on the activity of recorded areas within the same session.

A.13 Impact of loss terms on NeuroPaint performance (MAP dataset)

To evaluate the contribution of each designed loss term on real data, we compared the performance of different NeuroPaint variants on the MAP dataset. In line with the synthetic data results in Fig. 2 and Fig. S2, the inclusion of the consistency loss was essential for enabling NeuroPaint to outperform the LFADS baseline. In addition, NeuroPaint trained with all three loss terms performs the best. Surprisingly, NeuroPaint-Linear, a purely linear variant of the model, also performed competitively, comparably with the LFADS baseline, highlighting the strength of the overall NeuroPaint framework and its potential to reveal interpretable inter-areal communication among all areas of interest, including both recorded and unrecorded ones.

Interestingly, we found that the variant of NeuroPaint trained with reconstruction and regularization loss (but without consistency loss) exhibited unstable performance across datasets. For example, while it performed reasonably well on the original synthetic dataset (Fig. 2), its performance degraded substantially on the lower firing rate synthetic data (Fig. S2) and on the MAP dataset (Fig. S3. This suggests that, in the absence of a consistency constraint, regularization can sometimes hinder model

performance—possibly by over-constraining the latent space or penalizing useful variability. These observations underscore the stabilizing role of the consistency loss in guiding the latent representation, especially under more realistic data distributions.

A.14 Interpretable area-specific latent dynamics in the IBL dataset revealed by NeuroPaint

Similar to results shown in Fig. 4, here we examine the inferred latent factors for the IBL dataset. Unlike the latent factors for SC in the MAP dataset, the latent factors for MRN in the IBL dataset did not show strong trial-type modulations (Fig. S4A). However, in line with results for the MAP dataset, the inferred latent factors in the IBL dataset remain consistent across trials over sessions, regardless of whether a given area was recorded or unrecorded (Fig. S4A, B). We evaluated area-to-area representation similarity based on the inferred latent factors, across three trial periods, defined as three consecutive 0.5-second intervals after stimulus onset (Fig. S4C,D). Consistent with anatomical expectations, DG and CA1, both belonging to the hippocampus, exhibited high similarity with each other but not with other areas. In addition, two thalamic nuclei, PO and VPM, also exhibit high similarity across the three periods. Notably, the overall inter-areal similarity structure changed over time: although coherent sub-networks of areas remained present throughout the trial, they became more fragmented in the later periods, exhibiting both reduced similarity and a narrower set of similar areas. This dynamic change likely reflects a shift in task phase—from visual processing to decision-making and motor execution.

A.15 LFADS with larger architectures does not outperform NeuroPaint

To address the concerns regarding potential model capacity limitations in LFADS, we trained LFADS models on the MAP dataset with larger architectures (256 units in encoder, controller and generator). We tested the number of latent factors of 243 (matching the number of pooled NeuroPaint latent factors across areas) and 49 (matching the largest single-area NeuroPaint latent factors). To maintain training stability and prevent loss divergence, we reduced the learning rate from $3e^{-4}$ to $2e^{-4}$ here. Training converged successfully under this setting. The DFE for neurons in held-out areas, pooled over 40 sessions, is reported in Table 4. We found that NeuroPaint continued to outperform LFADS with larger architectures in terms of reconstruction accuracy. Interestingly, the higher-capacity LFADS models performed worse than the smaller model used in our main experiments, potentially due to overfitting given the increased number of parameters. These results suggest that the performance gap is not solely attributable to architectural capacity, and that the paradigm introduced in NeuroPaint is the key to its improved performance.

	**LFADS variants**			**NeuroPaint**
	27 factors, 100 units (used in Fig. 3G,H)	49 factors, 256 units	243 factors, 256 units	(used in Fig. 3G,H)
DFE	0.08 ± 0.006	-2.93×10^{20}	-9.12×10^{20}	$\mathbf{0.10 \pm 0.003}$
DFE (Worst 5% excluded)	0.10 ± 0.002	0.08 ± 0.002	-9.31 ± 1.31	$\mathbf{0.12 \pm 0.002}$

Table 4: Performance comparison across LFADS models of different capacities and NeuroPaint, evaluated by DFE (mean±s.t.e. over neurons in the held-out areas).

A.16 Calculating correlations between trial pairs of latent factors (MAP dataset)

To assess the consistency and trial-type-dependent modulation of inferred latent factors, we computed Pearson correlations between trial pairs of area-specific latent factors. All analyses were performed separately for each brain area.

Preprocessing. For each trial, the latent factor matrix was first mean-subtracted across time for each factor to remove the static offsets. The resulting matrix was then flattened into a vector by concatenating all factors.

Consistency results in the left block of Table 5. For each area, we computed Pearson correlations between flattened latent-factor vectors for every pair of trials, and grouped trial pairs into three categories: 1) Recorded - Recorded: both trials from sessions where the area is recorded 2) Unrecorded -

Table 5: Mean Pearson correlation between latent factors computed from trial pairs pooled across all sessions and averaged over brain areas (mean ± 1 standard deviation). See Appendix A.16 for calculation details.

**From all sessions**		**Trial-average subtracted, within a session**	
Trial pairs	Pearson's correlation	Trial pairs	Pearson's correlation
Recorded - Recorded	0.25 ± 0.05	Hit Left	0.59 ± 0.28
Unrecorded - Unrecorded	0.57 ± 0.10	Hit Right	0.59 ± 0.28
Recorded - Unrecorded	0.35 ± 0.06	Across Trial Type	0.53 ± 0.31

Unrecorded: both trials from sessions where the area is unrecorded 3) Recorded & Unrecorded: one trial from a session where the area is recorded, one from an unrecorded session.

For each area, we computed the averaged correlation within each group. The reported mean was obtained by averaging these per-area values across all areas, and the standard deviation reflects variability across areas.

Trial-type-dependent modulation results in the right block of Table 5. To control for the session-to-session variability, we perform the following analysis on trial pairs within each session, treating each session separately, and again also separately for each area.

For each session, we first computed the trial-averaged flattened latent factor by averaging over all *hit* trials. We then subtracted this average from each trial's flattened latent factor.

We calculated Pearson correlations between all pairs of trials within a session and grouped them into three categories, based on the trial types of the trial pair 1) Hit Left 2) Hit Right 3) Across Trial Type: one hit left and one hit right trial.

For each area and session, we averaged correlations within each group. The reported means and standard deviations were computed by averaging over sessions and areas.

A.17 Decoding choice and stimulus from NeuroPaint latent factors for held-out areas

To evaluate whether the latent factors capture the stimulus and behavioral variability, we performed decoding analysis on the MAP dataset. Specifically, we used logistic regression with cross-validation to decode either stimulus (high/low auditory tone) or choice (lick left/lick right/no lick) from the latent factors for each held-out area, and we compared the balanced accuracy to that decoding from held-out spike data (see Table 6). Stimulus was decoded using summed activity over the last 100 ms of the stimulus period; choice was decoded using summed activity over the last 100 ms of the delay period. Note that due to randomness, no data from VM-VAL was held out in the MAP dataset. As a result, we are unable to report results for this area.

We found that decoding accuracy for inferred latent factors is typically slightly higher than that from held-out spike data, suggesting that the inferred latent factors successfully capture the stimulus/behavioral variability in the brain area. This decoding improvement also suggests that the inferred latent factors provide a denoised and more complete representation than the held-out population spike data subsampled from the area of interest.

		ALM	lOrb	vlOrb	Pallidum	Striatum	MRN	SC
Stimulus (chance level= 0.5)	spike data	0.60 ±0.12	0.58 ±0.08	0.53 ±0.08	0.65 ±0.07	0.53 ±0.04	0.62 ±0.08	0.66 ±0.08
	latent factors	0.71 ±0.08	0.50 ±0.13	0.61 ±0.13	0.80 ±0.13	0.73 ±0.06	0.68 ±0.12	0.76 ±0.09
Choice (chance level= 0.33)	spike data	0.40 ±0.15	0.41 ±0.13	0.37 ±0.12	0.46 ±0.17	0.58 ±0.13	0.45 ±0.09	0.52 ±0.21
	latent factors	0.50 ±0.14	0.51 ±0.13	0.57 ±0.14	0.68 ±0.12	0.63 ±0.11	0.63 ±0.16	0.70 ±0.16

Table 6: Decoding accuracy from spike data or latent factors for the held-out areas with logistic regression, evaluated by balanced accuracy (mean±s.t.d. over sessions).

A.18 Intrinsic dimensionality of predicted firing rates for each brain area from NeuroPaint latent factors

In Table 7, we report the intrinsic dimension of the reconstructed firing rates based on the NeuroPaint latent factors. Specifically, for each recorded area in the MAP dataset, we calculated the participation ratio of the predicted firing rates, which quantifies the effective number of linear dimensions contributing to the variance. Notably, the number of latent factors we used is much larger than the intrinsic dimensionality of predicted firing rates, suggesting that similar performance may be achievable with fewer latent factors. We will explore this in future work.

	ALM	lOrb	vlOrb	Pallidum	Striatum	VAL-VM	MRN	SC
Number of latent factors	29	31	37	23	31	49	22	21
Intrinsic dimensionality (mean±s.t.d.)	4.71 ±1.45	4.82 ±1.40	4.21 ±1.43	3.51 ±1.36	4.19 ±1.53	5.63 ±1.40	3.90 ±0.98	4.50 ±1.05

Table 7: Intrinsic dimensionaliy of predicted firing rates reported against the number of latent factors from NeuroPaint for the MAP dataset.

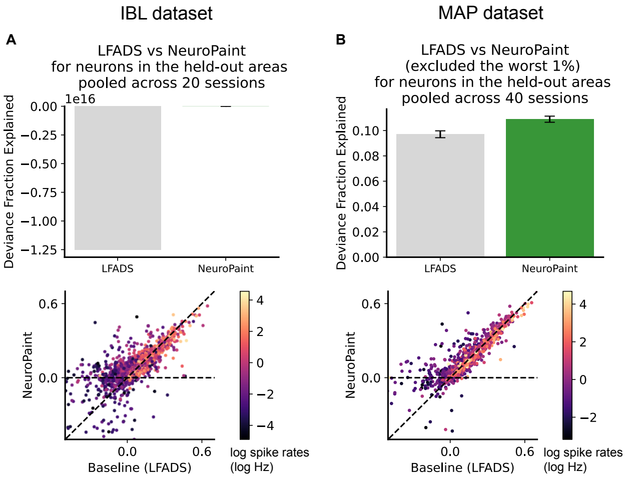

Supplemental Figure 1: Prediction performance for neurons from held-out brain areas across the two datasets. **(A)** Top: mean and standard error of DFE on the IBL dataset across all neurons, here LFADS yields a mean DFE on the order of $-1e16$, primarily due to a subset of neurons with extremely poor predictions; compare Fig. 3C where the 1% of neurons with the poorest predictions were excluded. Bottom: per-neuron DFE for all the held-out areas in 20 IBL sessions. **(B)** Top: mean and standard error of DFE on the MAP dataset, excluding the 1% of neurons with the poorest predictions; compare Fig. 3G, which included all neurons. Bottom: per-neuron DFE for all the held-out areas in 40 MAP sessions.

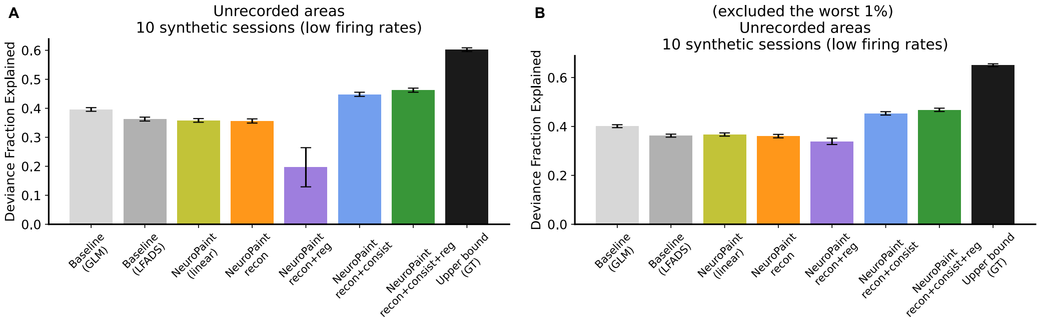

Supplemental Figure 2: Prediction performance from baselines, variants of NeuroPaint and ground truth firing rates for neurons from unrecorded areas in the synthetic dataset with lower firing rates. **(A)** Mean and standard error of DFE on the synthetic dataset with lower firing rates across all neurons. **(B)** Mean and standard error of DFE on the synthetic dataset with lower firing rates, excluding the 1% of neurons with the poorest predictions.

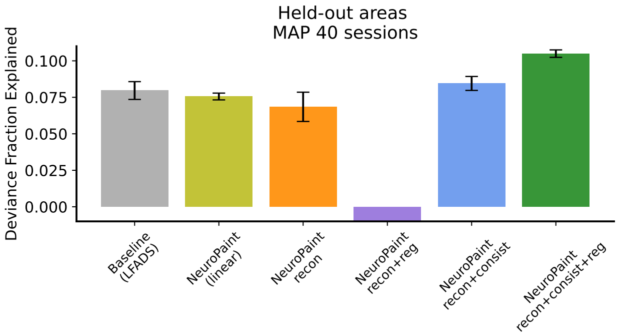

Supplemental Figure 3: Prediction performance from LFADS and variants of NeuroPaint for neurons from held-out brain areas in the MAP dataset. Bar plot shows mean and standard error of DFE on the MAP dataset across all neurons. For visuallization purpose, we truncate the y axis below –0.01. For NeuroPaint trained with reconstruction and regularization loss, its mean of DFE is -3.83, standard error of DFE is 0.11.

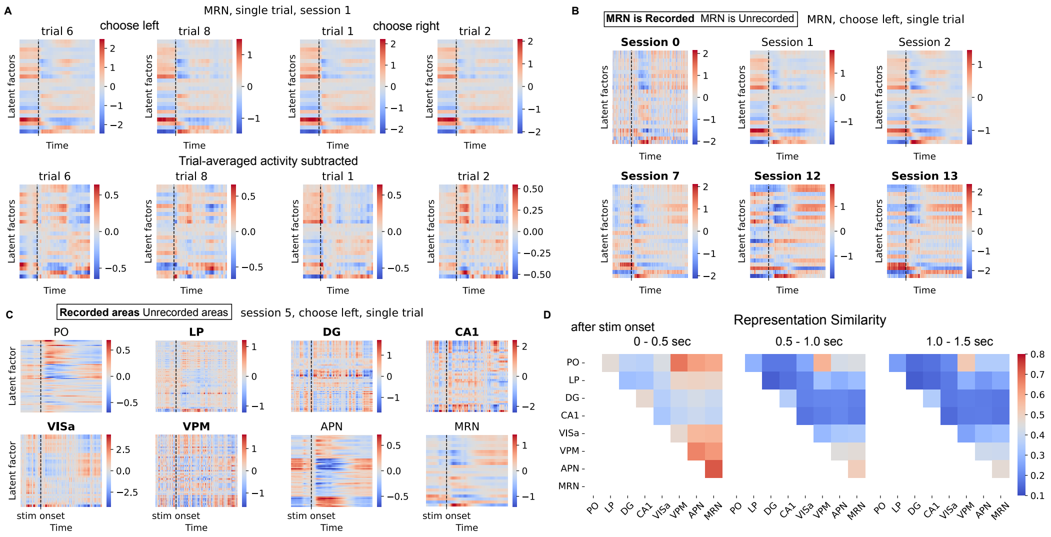

Supplemental Figure 4: *NeuroPaint's area-specific latent factors for the IBL dataset.* **(A)** Inferred latent factors for the Midbrain Reticular Nucleus (MRN), an unrecorded area in session 1 from the IBL dataset, during example trials across the *choose left* and *choose right* conditions. The bottom row shows the same latent factors with the trial-averaged activity subtracted. **(B)** Inferred single-trial latent factors for MRN (both recorded and unrecorded) across six sessions. **(C)** Latent factors for all eight areas (both recorded and unrecorded) in a *choose left* trial from a single session in the IBL dataset. **(D)** Representation similarity analysis (RSA) of latent factors across eight brain areas (recorded and unrecorded) for choose-left trials for three periods after stimulus onset in the IBL dataset, with values averaged across trials and pooled over sessions.
